# Quantitative Assessment of Voids’ Impact on Mechanical Properties of Standard Dogbone Model Versus End-User Component in Non-Linear Geometry

**DOI:** 10.3390/polym17070956

**Published:** 2025-03-31

**Authors:** Yasaman Mohseni, Sinduja Suresh, Marie-Luise Wille, Prasad K. D. V. Yarlagadda, J. Paige Little

**Affiliations:** 1 School of Mechanical, Medical and Process Engineering, Queensland University of Technology, Brisbane, QLD 4000, Australia; s.suresh@qut.edu.au (S.S.); m.wille@qut.edu.au (M.-L.W.); j2.little@qut.edu.au (J.P.L.); 2 Australian Research Council (ARC) Training Centre for Multiscale 3D Imaging, Modelling and Manufacturing (M3D Innovation), Queensland University of Technology, Brisbane, QLD 4000, Australia; y.prasad@unisq.edu.au; 3 Centre for Biomedical Technologies, School of Mechanical, Medical and Process Engineering, Queensland University of Technology, Brisbane, QLD 4000, Australia; 4 Biomechanics and Spine Research Group, Centre for Children’s Health Research, Queensland University of Technology, 62 Graham, South Brisbane, QLD 4101, Australia; 5 Max Planck Queensland Centre (MPQC) for the Materials Science of Extracellular Matrices, Queensland University of Technology, Brisbane, QLD 4000, Australia; 6 School of Engineering, University of Southern Queensland, Springfield, QLD 4300, Australia

**Keywords:** additive manufacturing, Fused Filament Fabrication technique, manufacturing defects (voids), standard dogbone model versus end-user component, mechanical properties, polymer

## Abstract

Additive manufacturing (AM) offers advantages such as design flexibility and reduced production times, but defects like voids impact mechanical performance and limit its broader adoption. This study quantitatively examines the relationship between void characteristics (volume fraction, distribution, and size) and mechanical properties in both linear and non-linear geometries, represented by a dogbone model and an end-use component, respectively. Samples were produced using Fused Filament Fabrication (FFF) with varying overlap levels to control void content. As the overlap increased from 0% to 99%, voids transitioned from large linear gaps to smaller point-shaped voids. In non-linear geometry, void reduction from 12% to 2% led to a threefold improvement in mechanical response, while in dogbone samples, voids decreased from 12% to nearly 0%, improving the elastic modulus by only 1.5 times. This disparity is due to differences in void distribution, as voids in non-linear geometries affect both margins and internal layers, significantly influencing structural integrity. The findings highlight the importance of the void location in determining mechanical performance and emphasize the limitations of using linear dogbone models to assess void–property relationships in complex 3D-printed structures.

## 1. Introduction

AM is a methodology employed for the production of physical 3D objects through the layer-by-layer construction of components based on a digital model [1,2,3,4]. Initially designed for small-scale production and prototyping, AM has had substantial growth in the past decade due to the advantages it offers over traditional fabrication methods [1,5]. Among the most prevalent AM techniques, Fused Filament Fabrication (FFF) or Fused Deposition Modelling (FDM) offers significant advantages in the automotive industry for polymeric components by enabling the cost-effective, low-volume production of lightweight and customized parts without extensive tooling [6,7,8]. This allows for faster production, optimized designs, and greater flexibility in meeting customer demands [9]. Although FFF printing offers various advantages, a significant challenge is the formation of voids within 3D-printed parts [10]. These voids, which occur during the layer-by-layer extrusion process, can reduce mechanical properties and overall product quality, weakening structural integrity [11,12]. Voids are primarily caused by low injection pressure and weak bonding between layers, leading to air pockets that compromise the part’s strength [13,14,15,16,17].

Another key factor in void formation is the temperature difference between the newly deposited filament and the previous layer [18]. Insufficient melting or a cooler previous layer can create gaps, resulting in voids [19]. A significant amount of research has focused on categorizing voids in FFF and investigating how different processing parameters, such as printing speed [20,21], layer thickness [22,23,24], extrusion temperature [21,25], and nozzle diameter [26], affect void formation. For example, higher printing speeds have been found to result in incomplete bonding between layers [21], while incorrect layer thickness and temperature settings can cause inconsistencies in material deposition, all of which contribute to void formation [20,27]. When evaluating the mechanical properties of FFF parts, two main factors are considered: the effects of printing and slicing parameters and the presence of uncontrollable voids. While many studies have examined variables such as infill density, layer height, and print speed in relation to part strength, research on the impact of void formation on mechanical performance remains limited [20,23,28,29]. Most investigations into voids have been conducted on 2D sections, either experimentally or numerically [30,31]. Some researchers have modelled layer adhesion with different raster angles, observing that the orientation between interlayer voids and the loading direction governs local stress distribution. Additionally, an increased layer height and decreased layer width result in more voids, leading to lower stiffness [32].

However, there is no report on void size and void fraction, except imaging of cross-sections showing the difference in voids’ size and shape for a single cross-section image [32,33]. Some researchers have explored various infill strategies and densities using SEM to examine the cross-sections of printed samples, finding that higher infill densities led to fewer tiny voids [32]. They suggested that this reduction in voids could explain the improved mechanical properties in these samples; however, these observations lacked quantitative analysis to validate their findings.

Existing studies often assess the effects of voids on static properties through 2D cross-sections, relying on qualitative observations from SEM without comprehensive measurements of void shape, dimensions, location, and distribution [8,25,34]. Despite findings that voids reduce part strength, these studies lack a detailed quantitative analysis to define which specific void characteristics are most influential and how [17,35,36].

Additionally, the majority of research studies have used standard models, such as dogbone models in the case of tensile tests, to study voids [37,38]. There is also the question of whether dogbone models remain a valid representative for this purpose. The dogbone model is composed of linear geometry, and it is uncertain whether dogbone specimens can reliably represent non-linear geometry for FFF parts. The sensitivity of parameters involved in creating voids, as well as the sensitivity of mechanical properties to voids, is influenced by geometry when changed from linear to non-linear. While some quantitative relationships between voids and mechanical properties have been suggested, there is a distinct lack of comprehensive studies that rigorously quantify these relationships for both linear and non-linear geometries. Curved, overhanging structures and holes introduce different types of voids in 3D space rather than just 2D, which will be significantly different from a dogbone with linear geometry. This difference has never been discussed in detail.

This study aims to quantitatively assess the correlation between voids and mechanical properties in both a standard geometry and an end-user component. This investigation aims to compare these relationships between the two models, evaluating how effectively the standard geometry reflects the associations between voids and mechanical properties observed in the end-user component.

Addressing this critical research gap, this study concentrates on an interior polymeric automotive component, the sunvisor holder clip, chosen for its frequent usage and the significant stress and degradation it endures, which results in elevated failure rates. Furthermore, this component was selected due to its complex overhang structures and intricate curves, which serve to exemplify the challenges faced by end-user components in the automotive industry. Additionally, this research used polypropylene (PP) as the material for 3D printing, a flexible polymer known for its excellent damping properties suitable for automotive applications, especially in interior design [39,40,41].

## 2. Materials and Methods

The study utilized a dogbone model, commonly used in research, as the standard geometry. Additionally, considering that one of the primary advantages of 3D printing in the automotive industry is its capability for low-volume production [42], a specific automotive component, namely the sunvisor holder clip, was chosen for this examination. This clip was originally produced by the now defunct Commodore Holder Company and is currently unavailable on the market, posing a unique manufacturing challenge. Given its frequent breakdown, there is a pressing need to utilize 3D printing for the low-volume, customized production of the clip with varying shapes and geometries for different car models.

Voids were deliberately introduced by adjusting the overlap percentages during the slicing stage, ranging from 0% to 50% to 99%, with the expectation of progressively increasing void incorporation within the models. This study investigated how overlap affects void fraction, size, and distribution in the models printed with different raster angles. Furthermore, a thorough quantitative analysis was conducted to assess the relationship between voids (fraction, size, and distribution) and tensile properties of the components. These findings were then compared between dogbone and sunvisor holder clip models to determine the transferability of results from the standard geometry to a more complex one.

### 2.1. CAD Model

**Figure 1:**
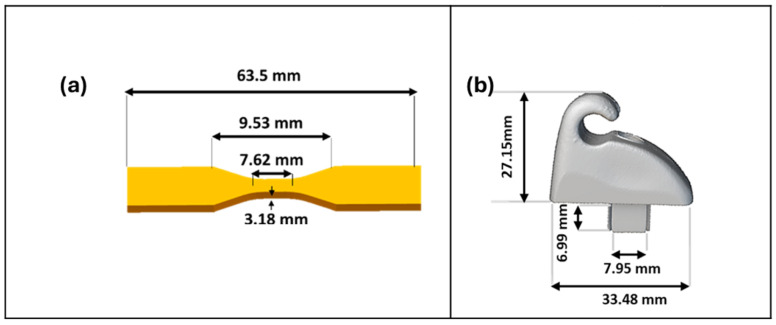
(**a**) The standard model. The CAD model of the dogbone is used as the standard shape according to ASTM D638. (**b**) The end-user automotive component. The sunvisor holder clip was conventionally manufactured by the now defunct Commodore Holder Company via injection moulding.

The selection of the dogbone shape adhered to the ASTM (American Society for Testing and Materials) standards, specifically ASTM D638 type V, which defines the specifications for plastic testing (Figure 1). The dogbone model was created in SolidWorks 2022, ensuring compliance with ASTM D638 [43], with a thickness of 3.18 mm.

The sunvisor holder clip, which was conventionally manufactured by the Commodore Holder Company and is no longer in production, was available only as a physical model. It was scanned using X-ray micro-computed tomography (Micro-CT) to ensure precise geometric measurement and accurate 3D shape reconstruction. The challenges posed by curved surfaces and overhang structures, which can hinder accurate measurement using conventional callipers, were addressed by employing Micro-CT technology (SCANCO Medical AG in Brütisellen, Switzerland) with a notably higher level of accuracy. The conventional component was carefully positioned on a specialized tube designed to accommodate the size and type of the holder (48 mm). The Micro-CT scanning parameters were set to 45 KVP for the voltage, 0.1 mm AL (PMMA) for the calibration, and AL 0.1 mm for the filter. The X-ray scan was conducted at a resolution of 14 µm (voxel size), ensuring detailed and precise imaging. All images were exported as DICOM files for 3D visualization and further analysis. Subsequently, the Avizo software (version 9.5.0) was employed to export the surfaces captured during the Micro-CT scan into STL file format.

### 2.2. Three-Dimensional Printing

**Figure 2:**
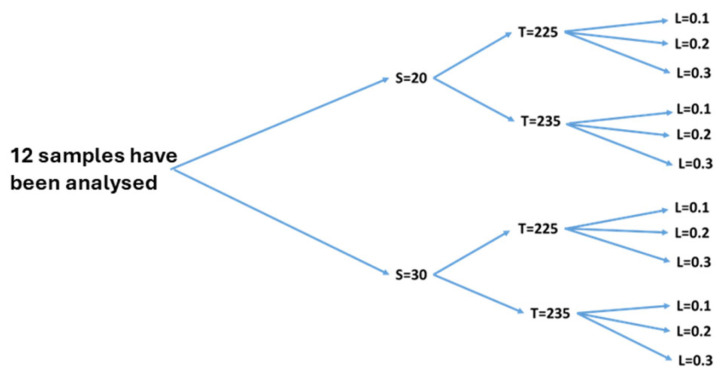
Twelve combinations have been analysed. Speed (S), temperature (T), and layer Thickness (L).

Polypropylene (PP) was chosen as the material for printing both the dogbone specimens and the sunvisor holder clips. Given the complexity of printing the clip, a range of printing parameters was tested to determine which layer thickness, nozzle speed, and nozzle temperature were appropriate for successful fabrication. Three different levels of layer thickness, two levels of nozzle temperature, and two levels of nozzle speed were tested (Table 1). These variations resulted in 12 unique combinations of printing parameters (Figure 2).

During the printing of the sunvisor holder clips with polypropylene, all 12 combinations of printing parameters were qualitatively assessed for print defects, such as layer delamination, incomplete extrusion, and deformation, in the curved surfaces and overhang structures of the component. This step was crucial, as the part does not have a simple geometry; it features complex curved surfaces and overhangs that require careful parameter selection to ensure manufacturability. This approach aimed to identify the most stable printing conditions for the study.

The bed temperature was consistently maintained at 80 °C, and the infill density was kept at 100%. Meanwhile, drawing from comprehensive literature reviews [4,5] the printing speed, extrusion temperature, and layer thickness were systematically varied [44].

**Figure 3:**
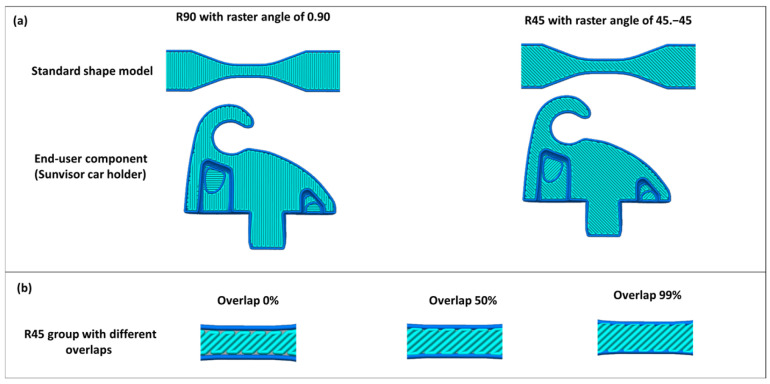
(**a**) Dogbone and sunvisor holder clip with three different raster angles. (**b**) R45 with three different overlaps.

Slicing parameters: In order to slice the model, two groups of raster angles of 0/90 and 45/45, namely R90 and R45 groups, were considered, respectively (Figure 3). Intentional voids were incorporated into the components by adjusting the overlap percentage in the slicing process, which changed from 0% to 50% to 99%.

### 2.3. Micro-CT and Voids Analysis

**Figure 4:**
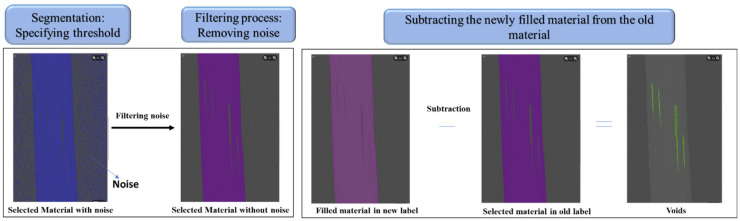
Different processes involved in detecting voids [45].

PP printed samples were subjected to Micro-CT analysis to characterize voids through porosity segmentation. Each experimental group underwent five replicate scans to ensure consistent and reliable results using the Micro-CT equipment (SCANCO Medical AG) at a resolution of 14 µm^3^, an electric potential of 45 KeV, and an Al 0.1 mm filter for both dogbone and clip samples. These scans generated 3D data, which were processed using reconstruction software. A volume of interest was selected by delineating contour lines to establish the threshold of scanned layers and identify regions of interest. Images were exported as DICOM files for 3D visualization. Avizo software (version 9.5.0) was used to detect and analyse defects. Figure 4 outlines the processes followed for void analyses.

In the Avizo analysis, an intensity threshold was selected to clearly separate the material pixels from the background. Filtering was applied to remove noise by selecting all connected pixels with similar intensity, thereby eliminating unconnected noise from the material. In the subsequent steps, a fully filled material was assigned a new label and subtracted from the previously filtered material to distinctly identify voids. The void fraction was then calculated using the following formula:

(1) $$Voids\;Fraction=\frac{Voids\;volume}{Material\;volume}$$

### 2.4. Mechanical Test

The mechanical testing setup was established using a tensile testing machine (Instron) with a load capacity of 5 kN.

**Figure 5:**
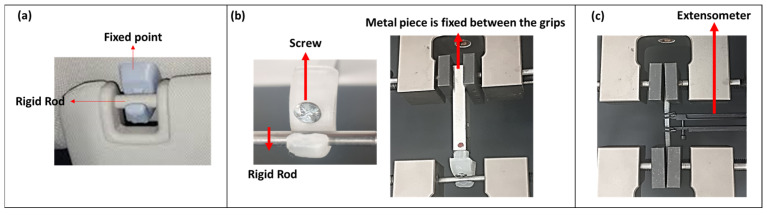
(**a**) The actual mechanism of the sunvisor holder clips. (**b**) The set up for conducting a tensile test on the sunvisor holder clips using the Instron machine. (**c**) The tensile test of the dogbone standard specimen using the Instron machine with an extensometer.

Sunvisor holder clip: Boundary conditions of the clip were replicated based on the real-world application. In reality, the bottom of the clips is fixed to the ceiling of the car, while a rigid rod is placed on the curved surface, connected to the clip (Figure 5a). In order to replicate these conditions in mechanical tests, a piece of metal was affixed to the bottom of the clip through a screw. The screw was inserted into the hole positioned at the centre of the clip and securely fastened to the metal piece (Figure 5b). To ensure stability, it was subsequently placed between two grips within the Instron machine. During testing, the sunvisor rod was either unclipped or pushed, resulting in the curved surface area opening by 3 mm, as illustrated in Figure 5b. As part of the evaluation process, a 5 mm displacement was applied at a speed of 5 mm/s to simulate real-world conditions and assess the component’s behaviour with a load capacity of 5 kN. Force–displacement curves were used to assess the mechanical response of the component under the defined loading conditions.

Dogbone: The samples were secured between the grips (Figure 5c). A mechanical extensometer was used to measure both strain and force. The tests were conducted at a speed of 5 mm/s, with five replicates. Data acquisition software (Bluehill Universal v3.0, Instron, United States) was utilized to collect strain and force data. To calculate stress based on the ASTM standard, the applied forces were divided by the cross-sectional area of the dogbone (Figure 5b). Young’s modulus, ultimate stress, and ultimate strain were determined from the stress–strain curves.

A normalization was introduced to compare the impact of voids on the mechanical response of the clip versus the dogbone to make the results comparable. This normalization involved dividing the voids fraction and mechanical properties of the samples with 50% and 99% overlap by the corresponding values observed at the reference point (0% overlap) (Equations (2) and (3)). The void fraction and mechanical properties of groups with 50% and 99% overlap were then analysed in relation to this reference point. By doing so, a normalized curve was generated, illustrating how the mechanical properties of the dogbone and clip change relative to the reference model as the voids fraction changes.

(2) $$\mathrm{Normalized}\;\mathrm{changes}\;\mathrm{in}\;\mathrm{void}\;\mathrm{volume}\;\mathrm{fraction}=\frac{VF-{VF}_{at\;0\%\;overlap}}{{VF}_{at\;0\%\;overlap}}\times 100$$

(3) $$\mathrm{Normalized}\;\mathrm{changes}\;\mathrm{in}\;\mathrm{mechanical}\;\mathrm{properties}=\frac{Mechanical\;propery-{Mechanical\;property}_{at\;0\%\;overlap}}{{Mechanical\;Property}_{at\;0\%\;overlap}}\times 100$$

## 3. Results

### 3.1. Selecting the Combination of Printing Parameters

All 12 parameter combinations were systematically assessed for printability during the fabrication process. Nine of the twelve combinations, specifically those involving a speed of 30 mm/s and a temperature of 235 °C, were deemed unsuitable due to printing failures. In these cases, the curved surfaces could not be accurately reproduced.

**Figure 6:**
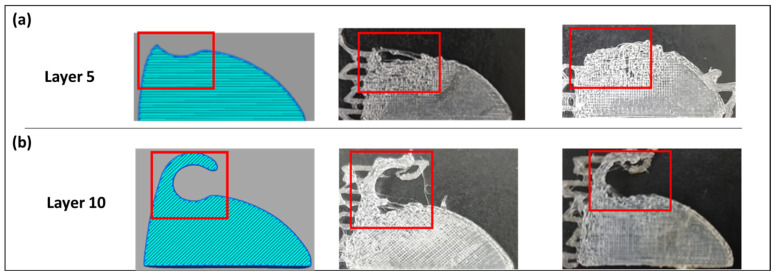
The top row (**a**): at a nozzle temperature of 235 °C, poor layer adhesion is observed, leading to incomplete bonding and structural instability in the curved region. The bottom row (**b**): at a printing speed of 30 mm/s, material deposition is inconsistent, resulting in deformation and incomplete layer formation, particularly in overhanging features.

Figure 6 illustrates the impact of printing temperature and speed on the manufacturability of the sunvisor holder clip, particularly at layers 5 and 10, where the curved surface begins to form. The left column shows sliced CAD models of the component, while the middle and right columns display actual printed samples at a speed of 30 mm/s and a temperature of 235 °C, with red boxes highlighting critical defect regions.

Notably, material deposition was unsuccessful at layers 5 and 10 of the print, leading to visible defects in the final structure

These failures indicated that temperatures above 225 °C led to insufficient cooling time between layers, impairing interlayer bonding (Figure 6a). Additionally, speeds exceeding 20 mm/s compromised the continuous material deposition, causing accumulation and deformation in curved regions (Figure 6b).

Subsequent analysis focused on three distinct combinations, all employing a print speed of 20 mm/s and a temperature of 225 °C, while varying the layer thickness. Layer heights of 0.2 mm and 0.1 mm demonstrated superior printing stability compared to 0.3 mm. Among the manufacturable combinations, the combination of 225 °C, 20 mm/s, and a layer height of 0.2 mm, which ensured stable printing conditions and minimized printing time, was chosen for fabricating the dogbone and sunvisor holder clips. To ensure experimental reliability, each printing parameter combination was repeated five times. For the qualitative printability assessment, a combination was considered successful only if all five repetitions exhibited a good print quality without visible defects. This approach ensured that the selected parameters consistently produced manufacturable parts.

### 3.2. Voids Analysis

Voids within the R45 and R90 groups were analysed using Micro-CT alongside image processing facilitated by Avizo 3D 2022.2.

#### 3.2.1. Standard Model (Dogbone)

**Figure 7:**
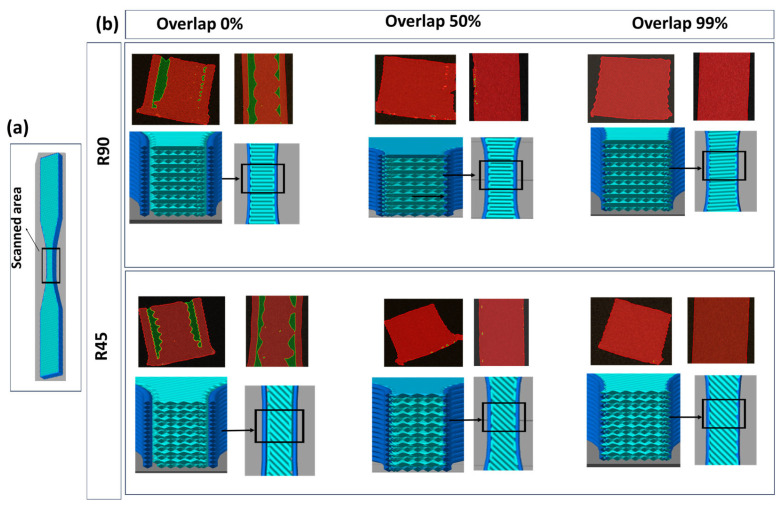
(**a**) The zone that was scanned for void analysis. (**b**) Void detection in R90 and R45 groups with different overlaps. The top images for each group show the scanned area by Micro-CT, and the images below show the corresponding slicing image.

Figure 7 provides insight into how different degrees of overlap influence the void occurrence within the R90 and R45 groups for the dogbone. As is clear from slicing images, the higher percentages of overlap result in closer proximity between the infill and marginal perimeter, thereby reducing the likelihood of void formation. Micro-CT analysis also confirms these observations, where for the groups featuring 0% overlap the integration between infill and perimeter is weak, with instances of non-integration observed in certain areas. However, as overlap increases, the infill infiltrates the perimeter, obviating the presence of gaps. The groups with 99% overlap show complete diffusion, yielding minimal point-dot-shaped voids across the sections.

**Figure 8:**
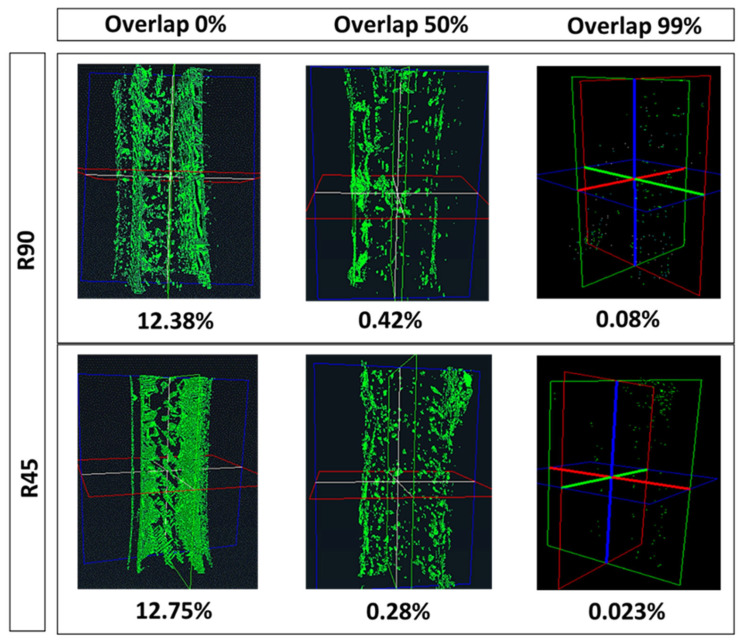
Scanned dogbones are compared alongside corresponding void volume fractions.

Figure 8 displays scanned images of the whole dogbone alongside corresponding void volume fractions. The group with 0% overlap exhibits the highest void fraction (roughly 13%), particularly evident around the edges of the dogbone. As the overlap increases, material layers form tighter bonds, and the void space reduces, transitioning toward a point-shaped morphology dispersed across the material. Elevating overlaps from 0% to 50% and 99% results in void reduction to roughly 0.2% and 0.04%, respectively.

**Figure 9:**
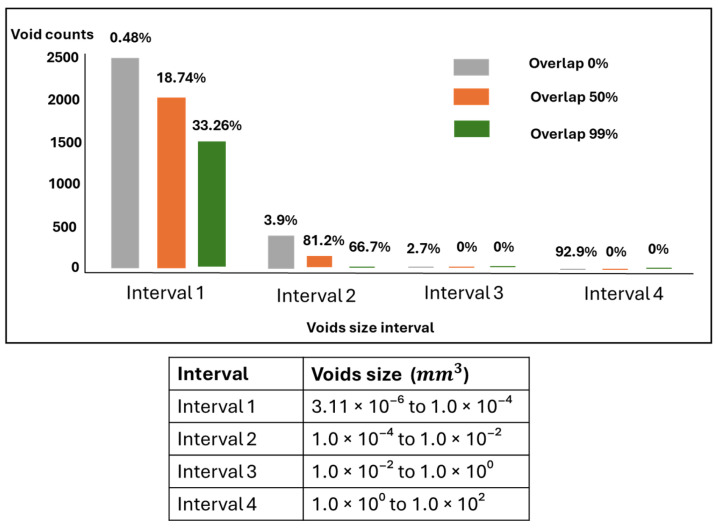
The distribution of voids in different sizes for the dogbone model. The void counts and their cumulative void volume across various ranges of void sizes have been reported. The percentage above each bar indicates the contribution of each void size range to the overall void volume within the structure.

Figure 9 illustrates how different ranges of void sizes contribute to the void volume fraction of the dogbone. It shows that the voids ranging from 3.11 × 10^−6^ to 1 × 10^−4^ mm^3^ contain the highest number of void counts but do not necessarily contribute the most to the overall void volume. In the case of the 0% overlap group, only 0.48% of the void volume is measured in this range, while 92.9% of the void volume is found in the range from 1.0 × 10^0^ to 1.0 × 10^2^ mm^3^, despite having only five void counts. As overlap increases, less disparity is observed across the void size distribution, where the most uniform void volume contribution is observed for the group with 99% overlap.

#### 3.2.2. End-User Model (Sunvisor Holder Clip)

**Figure 10:**
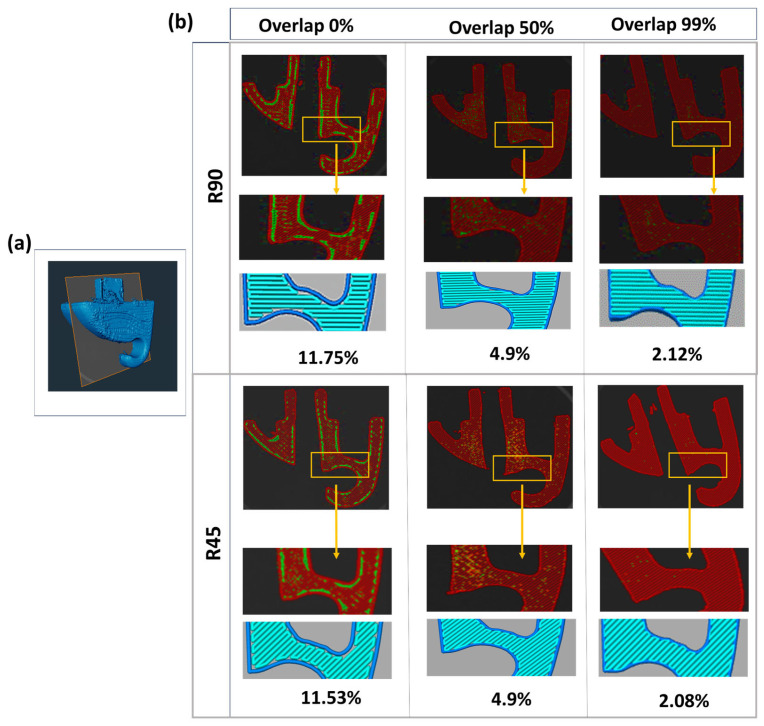
(**a**) The slicing and (**b**) Micro-CT images of the clip. Void detection for sunvisor holder clip in two groups of R90 and R45. Images below for each group show corresponding slicing images. The red areas represrnt the Micro-CT scan images, while the green spots indicate voids identifies at three different overlap levels. The blue regions correspond to the slicing images generated by the slicing software.

Figure 10 indicates the slicing and micro-CT images of the clip. As depicted, the intrinsic characteristics of curves, corners, and non-linear geometries within the clip model make it susceptible to the occurrence of voids around the margin perimeters. Interestingly, as the overlap increases from 0% to 99%, the void within the margin, as well as the internal regions, reduces, transitioning from large linear voids into minute point-shaped voids. This observation indicates that the quality of adhesion in margin areas directly impacts the quality of fusion between internal layers. This dependency can be attributed to the fact that weak adhesion with the perimeter in the margin can result in unstable polymer deposition as the nozzle travels non-linear paths. Therefore, low printing quality in non-linear margins results in low printing quality within infill layers. The void analysis indicated that the void fraction for both the R45 and R90 groups remained relatively close across different percentages of overlap, with a reduction trend from approximately 12% to 4.9% and 2.1% as overlap increased from 0% to 50% and to 99%, respectively.

**Figure 11:**
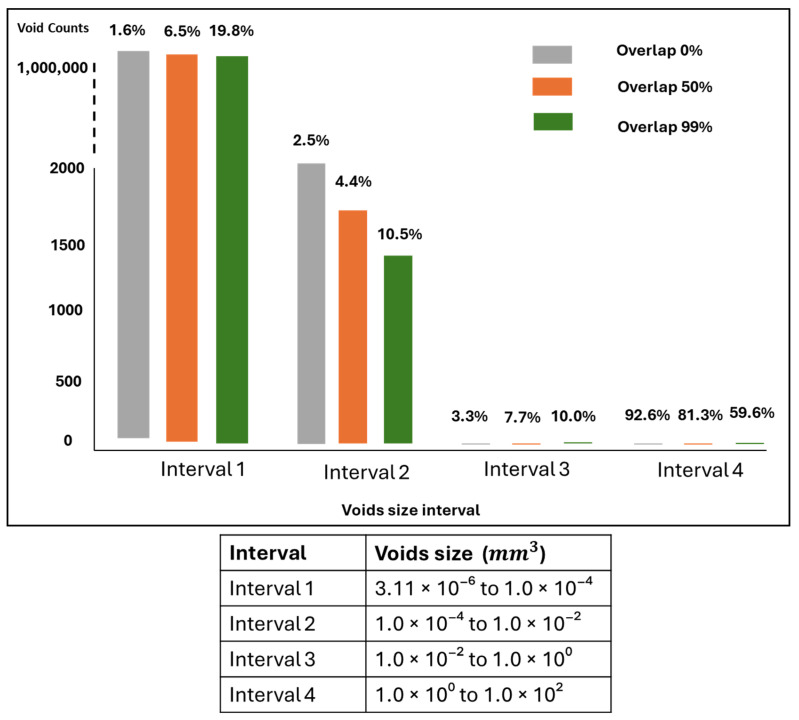
The distribution of voids in different sizes for the sunvisor holder clips. The void counts and their cumulative void volume across various ranges of void sizes have been reported. The percentage above each bar indicates the contribution of each void size range to the overall void volume within the structure.

Figure 11 illustrates the distribution of voids across different sizes. Voids in the range of 3.11 × 10^−6^ to 1.0 × 10^−4^ mm^3^ were the most frequent across the three groups, but their impact on the total void volume differed some groups had more significant contributions from larger voids beyond this range. The biggest voids range from 1.0 × 10^0^ to 1.0 × 10^2^ mm^3^ and indicate the highest contribution in overall void volume despite their lowest numbers in counts. As the overlap decreases, the disparity in void size becomes more uniform, and the volume contribution across different void sizes also decreases.

#### 3.2.3. Standard Model vs. End-User Model from Voids Analysis Perspective

**Figure 12:**
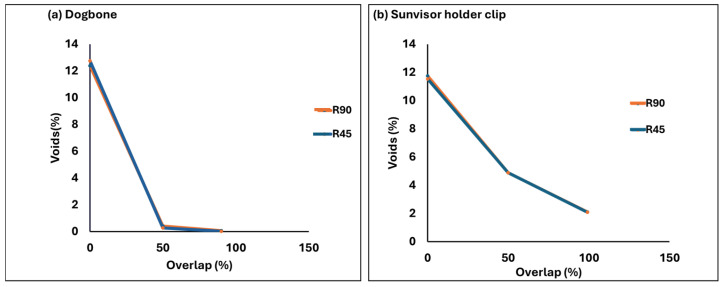
The voids change relative to the overlap for both dogbone and sunvisor holder clips.

For both the dogbone and clip, as the overlap increases, the voids are reduced; however, the alteration of the overlap from 0% to 50% results in a more rapid reduction in voids compared to the transition from 50% to 99% (Figure 12). This reduction is quantitatively more significant in the dogbone due to its simpler geometry. In the case of the clip model, voids manifest as a result of the combined influence of overlap settings and the inherent complexity of the geometry. With incremental increases in overlap, a gradual reduction in voids is observed at both the margin and internal layers of the component; nevertheless, even at a 99% overlap, a residual presence of approximately 2% voids with a size up to 1.0 × 10^1^ mm^3^ persists. Conversely, the dogbone geometry, characterized by its simplicity, demonstrates a more rapid decline in voids as the overlap is increased, where at an overlap of 99%, the prevalence of voids in the dogbone geometry nears insignificance with void sizes less than 1.0 × 10^−2^ mm^3^. These observations underscore the pivotal role of geometry complexity and its interaction with overlap parameters in governing the extent of void reduction.

Another highlighted difference is the direct impact of voids around the margins on the occurrence of voids between internal layers of the clip model, a phenomenon which was not observed for the dogbone. For the clip model, as the overlap increases, the voids around the margins as well as within the internal layers are reduced since the quality of fusion around the non-linear perimeter influenced the stability of polymer deposition and the occurrence of voids within internal layers. This dependency of internal voids on margin voids in the non-linear geometry was not observed for the standard model, thereby underscoring the reliability of the standard model in studies related to voids.

### 3.3. Mechanical Analysis

#### 3.3.1. Standard Model (Dogbone)

**Figure 13:**
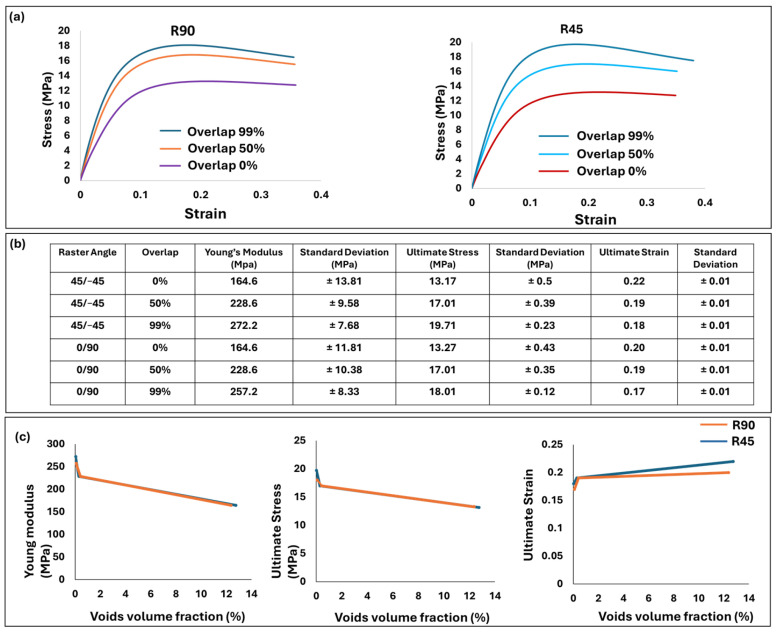
Mechanical properties of dogbone with different raster angles and overlap. (**a**) Stress–strain diagrams. (**b**) Young’s modulus, ultimate stress, and ultimate strain. (**c**) Comparison of void volume fraction and mechanical properties for both raster angles.

Figure 13a illustrates the mechanical properties of the dogbone at various percentages of overlap. The R90 and R45 groups showed closely comparable patterns of mechanical properties, which align logically with their similar isotropic properties and comparable void volume fractions. At a 99% overlap, both R45 and R90 exhibited the highest values of Young’s modulus, ultimate stress, and ultimate strain, with these values decreasing as the overlap decreased to 0% (Figure 13b).

Figure 13c depicts the relationship between mechanical properties and the void volume fraction. An increase in voids’ volume fraction from 0.04% to 0.2% resulted in a sharp decrease in mechanical properties, followed by a smoother reduction as the void volume increased from 0.2% to 13%. This highlights that dogbones with smaller void volume fractions exhibit greater sensitivity to voids, affecting their mechanical properties more significantly.

#### 3.3.2. End-User Model (Sunvisor Holder Clip)

**Figure 14:**
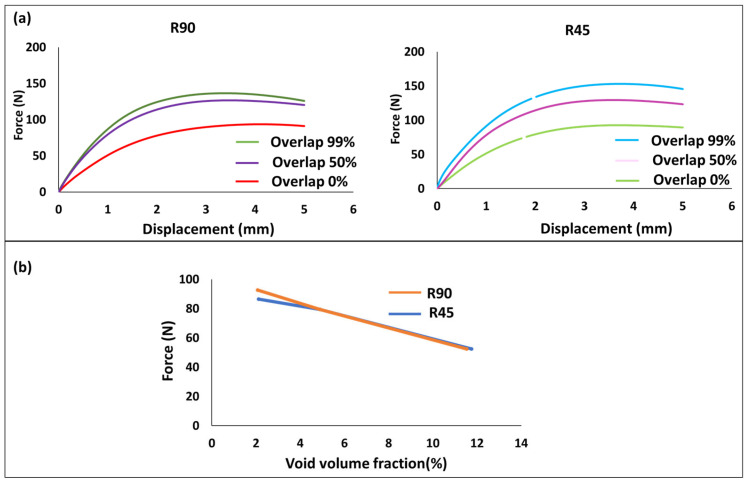
Mechanical properties of sunvisor holder clip at different raster angles and overlaps. (**a**) Force-displacement diagrams (**b**) Force corresponding to 1 mm displacement versus voids volume fraction.

The force and the corresponding displacement for the clip were reported in Figure 13a. It is observed that for R45 and R90, increasing the percentage of overlap improves the overall mechanical properties of the clip. In Figure 14b, the force required for a 1 mm displacement serves as a benchmark for comparing the mechanical properties of clips with different overlaps. A direct correlation between voids and mechanical properties, showing a linear relation, is observed for R45 and R90. At a 99% overlap, where voids constitute approximately 2%, the maximum force required for 1 mm displacement is around 90 N. Conversely, at a 0% overlap with voids at roughly 11%, the minimum force needed for 1 mm displacement is approximately 52.57 N, showcasing a linear response.

#### 3.3.3. Standard Model vs. End-User Model from Mechanical Perspective

The mechanical responses of the dogbone (elastic modulus) and the sunvisor holder clip (applied force for 1 mm displacement) exhibited distinct sensitivities to voids. To enable a direct comparison between the dogbone and the clip, normalization was introduced in Section 2.4. The 0% overlap group, where both the dogbone and the clip have a similar void volume fraction of approximately 12%, was used as the baseline reference. The void fraction and mechanical properties of the 50% and 99% overlap groups were then analysed relative to this reference point.

**Figure 15:**
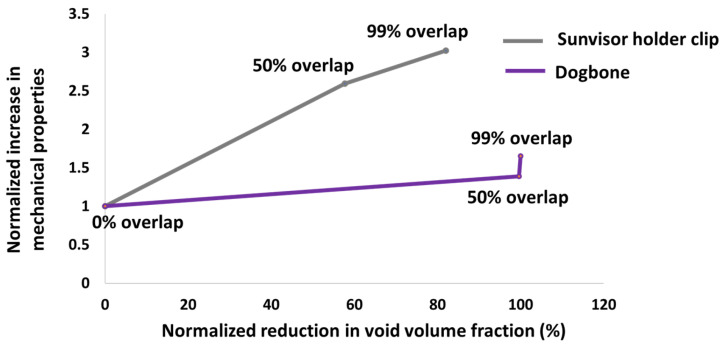
Normalized increase in mechanical properties—elastic modulus for dogbone specimens and applied force at 1 mm displacement for sunvisor holder clips—versus normalized reduction in void volume fraction for both models. Normalization provides comparable benchmark for evaluating effect of void reduction on mechanical performance in these two geometries.

In the case of the dogbone model, as the overlap varied from 0% to 99%, the voids volume fraction changed by 99%, accompanied by a 1.5-fold increase in the elastic modulus of the dogbone (Figure 15). In the case of the sunvisor holder clip, the void volume fraction changed by 85%, resulting in a 3-fold increase in the force required to move the clip by 1 mm (Figure 15). Although, at the 99% overlap, the clip still retained around 2% voids, with voids extending to 1.0 × 10^2^ mm^3^, while the dogbone had only 0.04% voids, with voids smaller than 1.0 × 10^−2^ mm^3^, the clip showed a sharper increase in mechanical response. This emphasizes that the mechanical properties of the clip are more sensitive to changes in voids. Therefore, the relationship between voids and the reduction in mechanical response observed in the dogbone underestimates the impact of voids in the clip and does not serve as a suitable representative model. Moreover, despite the non-linear geometry of the clip, it follows a more linear response when compared to the dogbone.

## 4. Discussion

This study demonstrates that increasing the overlap significantly reduces voids and enhances mechanical properties in both linear and non-linear geometries. Also, changing the overlap from 0% to 50% results in a more significant reduction in voids compared to the change from 50% to 99%.

In linear geometry, increasing the overlap from 0% to 99% rapidly decreases the void volume fraction, from 12% to nearly 0%, with void sizes reduced to less than 1.0 × 10^−2^ mm^3^. However, for non-linear geometry, increasing the overlap decreases the void fraction from 12% to 2% with the void size of 1.0 × 10^2^ mm^3^. This persistence of voids in non-linear geometries is less influenced by overlap and more related to the inherent complexity of the 3D printing process. This complexity underscores the substantial difference in the relationship between voids–mechanical properties when comparing the end-user component with non-linear geometry to linear dogbone geometry.

Despite the presence of 2% voids in the clips, the mechanical properties and the force required to move the component by 1 mm were increased 3-fold. While for the dogbone, despite reaching nearly 0% voids, the elastic modulus improved by only 1.5-fold. This highlights the greater sensitivity of non-linear geometry to void variations compared to linear geometry.

The disparity in sensitivity can be attributed to the different ways voids are distributed within these geometries. In the clips (non-linear model), changes in overlap influence voids not only at the margins but also within internal layers, which can substantially impact structural integrity and potentially cause stress concentrations. While in the dogbone (linear model), voids predominantly occur at the margins, resulting in a less pronounced effect on mechanical performance. This emphasizes the critical role of void location, particularly within infill layers, in determining mechanical behaviour. Given that the majority of research has focused on linear dogbone geometry when studying voids, or even the impact of voids on mechanical responses [46,47,48], these findings are not easily transferable to end-user components with non-linear geometries. Considering the heightened sensitivity of models to voids within infill layers, the dogbone model emerges as a less desirable standard for 3D-printed parts.

The material used in this research is PP, which offers special anti-vibration characteristics and enhanced flexibility [39,40]. However, despite the benefits PP offers, its integration into 3D-printed automotive components has been limited, likely due to challenges in the printing process and its susceptibility to void formation [49,50,51,52]. This research demonstrates stable printing and slicing conditions for PP to minimize voids and evaluates void formation and its impact on mechanical properties using examples of standard dogbone geometry and end-user components with non-linear geometry. These findings could facilitate a broader utilization of PP in 3D printing technology within the automotive industry.

## 5. Conclusions and Future Work

Dogbone samples with a linear geometry exhibit a rapid reduction in void volume, reaching nearly 0% as the overlap increases to 99%. However, this reduction results in only a 1.5-fold improvement in the elastic modulus. In contrast, sunvisor holder clips with a non-linear geometry, despite retaining 2% voids at the 99% overlap, demonstrate a 3-fold increase in mechanical response.

This difference is attributed to void distribution: in non-linear geometries, voids near the margins significantly affect both external and internal layers, altering the structural integrity. Increasing the overlap reduces voids not only at the margins but also within the internal layers, enhancing mechanical performance more significantly in non-linear geometries than in linear dogbone specimens.

These findings emphasize the critical role of geometric complexity in void distribution and highlight the limitations of using dogbone specimens to predict mechanical behaviour in real-world components. The results indicate that non-linear geometries are more sensitive to void content and distribution, reinforcing the need for application-specific assessments in additive manufacturing.

Quantitative analysis of the relationships between voids and mechanical proeprtiesvoids–mechanical properties is crucial for the quality control of 3D-printed parts. Traditional quality control methods, which often involve the destructive testing of randomly selected samples, are impractical for 3D-printed parts, particularly in low-volume production, where defects are more prevalent and a larger number of samples should be tested. Voids–mechanical studies pave the way for developing non-destructive quality control methods for 3D-printed components by providing an understanding of the quantitative relationships between voids and mechanical functionality. This enables the possibility of quality control through voids inspection rather than relying on destructive testing.

Another key mechanical property of the clip as an interior automotive component is fatigue. Future research will delve into comprehensive quantitative assessments that directly link void characteristics to fatigue behaviour, with the goal of understanding the precise relationship between void volume and its impact on fatigue properties. Furthermore, this research will explore whether the dogbone (linear geometry) can reliably represent end-user components with non-linear geometries when assessing dynamic properties.

## Figures and Tables

**Table 1:** Parameters optimized for printing dogbone and sunvisor holder clip.

Experiment NO	S = Printing Speed (mm/s)	T = Nozzle Temperature (°C)	L = Layer Thickness (mm)	Combination
1	20	225	0.1	(20,225, 0.1)
2	20	225	0.2	(20,225, 0.2)
3	20	225	0.3	(20,225, 0.3)
4	20	235	0.1	(20,235, 0.1)
5	20	235	0.2	(20,235, 0.2)
6	20	235	0.3	(20,235, 0.3)
7	30	225	0.1	(30,225, 0.1)
8	30	225	0.2	(30,225, 0.2)
9	30	225	0.3	(30,225, 0.3)
10	30	235	0.1	(30,235, 0.1)
11	30	235	0.2	(30,235, 0.2)
12	30	235	0.3	(30,235, 0.3)

## Data Availability

The data presented in this study are available on request from the corresponding author.
